# Sustaining momentum in gender equity: Lessons from the women in European Society of Sports Traumatology, Knee Surgery and Arthroscopy initiative

**DOI:** 10.1002/ksa.70157

**Published:** 2025-10-27

**Authors:** Lorenz Pichler, Laura De Girolamo, Elizabeth Arendt, Berte Bøe, Mette Renate Andersen, Pim van Dijk, Guri Ranum Ekås, Elizaveta Kon, Maristella Francesca Saccomanno, Katja Tecklenburg

**Affiliations:** ^1^ Department of Orthopedics and Trauma‐Surgery Medical University of Vienna Vienna Austria; ^2^ Center for Musculoskeletal Surgery Charité—Universitätsmedizin Berlin Berlin Germany; ^3^ Orthopaedic Biotechnology Laboratory IRCCS Ospedale Galeazzi Sant'Ambrogio Milan Italy; ^4^ Department of Orthopaedic Surgery University of Minnesota Minneapolis USA; ^5^ Division of Orthopaedic Surgery Oslo University Hospital Oslo Norway; ^6^ Aleris Hospital Tromsø Tromsø Norway; ^7^ Department of Orthopaedic Surgery and Sports Medicine and Academic University Medical Center University of Amsterdam Amsterdam The Netherlands; ^8^ Academic Center for Evidence‐based Sports Medicine, Academic University Medical Center University of Amsterdam Amsterdam The Netherlands; ^9^ Orthopaedic Department Akershus University Hospital Nordbyhagen Norway; ^10^ University of Oslo Oslo Norway; ^11^ Oslo Sports Trauma Research Centre Oslo Norway; ^12^ Department of Biomedical Sciences Humanitas University Milan Italy; ^13^ IRCCS Humanitas Research Hospital Milan Italy; ^14^ Department of Orthopedics Fondazione Policlinico Universitario A. Gemelli, IRCSS Rome Italy; ^15^ Medalp Sportclinic Imst Austria

**Keywords:** gender equality, gender equity, orthopaedics, women in ESSKA

## Abstract

**Abstract:**

Women remain underrepresented in orthopedic surgery, accounting for only 13% of surgeons in Europe despite forming the majority of physicians overall. Structural barriers, including limited mentorship and work–life challenges, continue to hinder progress. To address these issues, the European Society of Sports Traumatology, Knee Surgery and Arthroscopy (ESSKA) launched the Women in ESSKA initiative in 2018, fostering mentorship, leadership and visibility. Female membership and congress participation have since more than doubled, reflecting steady progress. Sustained equity efforts must extend beyond participation numbers to include transparent leadership pathways and fair representation in authorship and decision‐making. True progress will depend on collaboration between women and men alike, ensuring that gender equity in orthopedics strengthens, not replaces, the core value of merit‐based excellence.

**Level of Evidence:**

Level V.

**Abbreviations**
DEI: Diversity, equity and inclusion
ESSKA: European Society of Sports Traumatology Knee surgery and Arthroscopy
EU: European Union
LGBTIQ: Lesbian, Gay, Bisexual, Transgender, Intersex, and Queer
U.S.: United States

## DIVERSITY, EQUITY AND INCLUSION

DEI initiatives in state institutions, private companies, and public schools and universities have recently witnessed significant pushback in the United States, including budget cuts, workforce reduction, and even anti‐DEI legislation [1, 22].

In contrast, the European Union (EU) has introduced several action plans and equality strategies addressing key DEI areas such as anti‐racism, LGBTIQ rights, and gender equity [6, 7, 8]. Specifically, the EU roadmap for women's rights emphasizes ‘… an urgent need to reiterate, reaffirm and reinforce the commitment to gender equality…’ [9].

In health care, women constitute the majority of the global work force (67%), and specifically more than half of physicians in Europe (53%) [6] (2019). Despite this, women remain significantly underrepresented in orthopedics, making up only 13% of orthopedic surgeons across Europe (median; range: 1% in Turkey to 27% in Germany) [3] (2020). A study published in *The Lancet* explored reasons why women leave surgical training, confirming previously reported factors such as a lack of role models, as well as structural limitations such as challenges associated with parenthood and parental leave policies, and the ergonomic design of surgical training environments [11, 13, 17, 30]. Other reports also indicate that 74% of female orthopaedic surgeons encounter episodes of subtle and/or unintentional discrimination during training, most commonly from patients or colleagues, causing additional challenges in their professional pathway and barriers to retention and advancement [3, 24].

## WOMEN IN ESSKA

The Lancet study also highlighted the ‘absence of interactions with Women in Surgery section and other supports’ [17] as an additional factor contributing to women leaving surgical training. To address the cultural barriers and above all to provide a tailored support, in 2018 the ESSKA launched the *Women in ESSKA* initiative. The initiative aims to build a network among women in orthopaedics to facilitate mentorship, educational and scientific collaboration and recruitment of female students to orthopaedic surgery and research [1].

Early exposure to orthopedics through programs such as the Perry Initiative and the Nth Dimension, addressed at medical students, has been shown to significantly increase the likelihood of women applying for and matching into orthopedic residency programs [10, 21].

Encouraging data report that in the United States, the percentage of female medical students applying to orthopedic residency programs has nearly doubled over the past 15 years, increasing from 11.8% in 2007 to 23.0% in 2022 [23]. In addition, targeted mentorship programs for women have also demonstrated self‐reported academic competencies, and program retention rates [14, 26, 28, 31]. Nevertheless, despite these positive trends, the overall visibility of women in orthopedics and particularly in leadership, academic authorship, and professional representation, has not changed substantially, underscoring the persistent structural and cultural barriers within the field. Only 8% of department chairs in surgery and 10% in orthopedic surgery in the United States are women, and 9% of editorial board members in leading international orthopedic journals are female [15, 18] (2024, 2021). The same trend is observed in professional medical societies, where women account for 40% of presidents across European medical societies in general, but only 8% in surgical societies [20, 27] (2025, 2019). Nonetheless, professional societies can play a vital role in developing female leadership by offering structured programs focused on leadership skills and career advancement strategies [16]. Such initiatives may also include transparency in committee selection, leadership pathways and societal awards [12].

## BEYOND NUMBERS: PROGRESS AND PERSPECTIVES

When the Women in ESSKA initiative was launched in 2018, only 5.5% of ESSKA members were women. By 2024, this share had more than doubled to 12%, and the proportion of fellowship applications submitted by women rose from 3.2% to 6.7%. Although a direct causal link between the initiative and these increases cannot be definitively established, this trend is both positive and encouraging.

Apart from activities in professional societies, participation in scientific conferences is another important factor to promote academic advancement and career progression among physicians [25]. An interesting study shows that in 10 major orthopaedic conferences in 2021, women represented only 12.6% of faculty and 58.5% of panels were male‐only, despite similar academic qualifications between genders [29] (2023). At the biannual ESSKA Congress, female representation increased from 7% of session chairs and 11% of speakers in 2014 to 13% in both roles by 2024. This positive trend has developed in parallel with the implementation of the Women in ESSKA initiative, which may have contributed to greater visibility and participation, although other broader cultural and institutional factors are also likely to play a role. While session chair appointments can be directly influenced through equity‐focused programming by organizing committees, the proportion of female speakers, largely dependent on abstract submissions, requires more indirect strategies, such as mentorship and visibility initiatives. ESSKA is committed to systematically monitoring women's participation in its scientific programs, and these data will be regularly reported in future articles published in ESSKA journals.

However, inclusion must not end at participation. Future metrics should aim beyond headcounts and assess the presence of women in authorship of clinical guidelines, editorial boards, and keynote speakers [19]. Such benchmarks will help ensure that diversity translates into meaningful influence within the field and in this regard equality and equity must not be championed by women alone but also through the active engagement of male allies in such programs [32]. Many male colleagues will embrace gender‐equality initiatives wholeheartedly when it is clear the ‘game’ is fair: positions are earned on merit, with candidates—women and men—judged by transparent, objective criteria and equal qualifications. The aim of equity efforts is not preferential selection because of gender, but the removal of structural barriers. Tokenism undermines trust and devalues achievement; rigorous, open processes build confidence that every appointment reflects excellence. In short, equity and merit are not competing goals—they are mutually reinforcing.

## SUSTAINING PROGRESS THROUGH PERSISTENCE

The growth in female ESSKA members and participation at the ESSKA congresses clearly demonstrates the impact of focused initiatives on gender equality and equity in orthopedic surgery. However, the progress made in recent years through such initiatives is often overestimated, leading to a decline in perceived need and support [5]. This ‘illusion of equity’ poses a risk to momentum, especially when initiatives are seen as having fulfilled their purpose once a certain numerical target is met.

Therefore, if the full potential of true gender equality and equity in orthopedic surgery is to be realized, we must remain persistent in promoting them. We need to acknowledge structural barriers, expand representation across career levels, and embed leadership development into the strategic goals of our societies.

However, it is important to acknowledge that behind every advancement, there are women that need to manage family life, surgical training, and structural inequities. Here, programs like Women in ESSKA can be extremely helpful, both by providing networking and mentoring opportunities as well as by promoting open and honest hiring procedures, inclusive conference schedules, and support systems that enable women to pursue challenging surgical careers.

Promoting equity means more than just opening doors; it requires creating an environment where women and men work side by side, advancing together, sharing leadership, and shaping the future of orthopedics as true partners. Progress in this field will come not from division but from collaboration between sexes, as exemplified by the Women in ESSKA initiative, which was conceived and continues to be actively supported by many male orthopedic surgeons together with their female colleagues.

## AUTHOR CONTRIBUTIONS

Each named author has substantially contributed to conducting the underlying research and drafting this manuscript.

## CONFLICT OF INTEREST STATEMENT

The authors declare no conflicts of interest.

## ETHICS STATEMENT

The authors have nothong to report.

## Data Availability

The data that support the findings of this study are available from the corresponding author upon reasonable request.
